# Peroxisome proliferator‐activated receptor γ coactivator 1‐α overexpression improves angiogenic signalling potential of skeletal muscle‐derived extracellular vesicles

**DOI:** 10.1113/EP090874

**Published:** 2022-12-01

**Authors:** Chris K. Kargl, Brian P. Sullivan, Derek Middleton, Andrew York, Lundon C. Burton, Jeffrey J. Brault, Shihuan Kuang, Timothy P. Gavin

**Affiliations:** ^1^ Department of Health and Kinesiology Max E. Wastl Human Performance Laboratory Purdue University West Lafayette IN USA; ^2^ Indiana Center for Musculoskeletal Health Department of Anatomy Cell Biology & Physiology Indiana University School of Medicine Indianapolis IN USA; ^3^ Department of Animal Sciences Purdue University West Lafayette IN USA

**Keywords:** angiogenesis, endothelial cells, extracellular vesicles, PGC‐1α, skeletal muscle

## Abstract

Skeletal muscle capillarization is proportional to muscle fibre mitochondrial content and oxidative capacity. Skeletal muscle cells secrete many factors that regulate neighbouring capillary endothelial cells (ECs), including extracellular vesicles (SkM‐EVs). Peroxisome proliferator‐activated receptor γ coactivator 1‐α (PGC‐1α) regulates mitochondrial biogenesis and the oxidative phenotype in skeletal muscle. Skeletal muscle PGC‐1α also regulates secretion of multiple angiogenic factors, but it is unknown whether PGC‐1α regulates SkM‐EV release, contents and angiogenic signalling potential. PGC‐1α was overexpressed via adenovirus in primary human myotubes. EVs were collected from PGC‐1α‐overexpressing myotubes (PGC‐EVs) as well as from green fluorescent protein‐overexpressing myotubes (GFP‐EVs), and from untreated myotubes. EV release and select mRNA contents were measured from EVs. Additionally, ECs were treated with EVs to measure angiogenic potential of EVs in normal conditions and following an oxidative stress challenge. PGC‐1α overexpression did not impact EV release but did elevate EV content of mRNAs for several antioxidant proteins (nuclear factor erythroid 2‐related factor 2, superoxide dismutase 2, glutathione peroxidase). PGC‐EV treatment of cultured human umbilical vein endothelial cells (HUVECs) increased their proliferation (+36.6%), tube formation (length: +28.1%; number: +25.7%) and cellular viability (+52.9%), and reduced reactive oxygen species levels (−41%) compared to GFP‐EVs. Additionally, PGC‐EV treatment protected against tube formation impairments and induction of cellular senescence following acute oxidative stress. Overexpression of PGC‐1α in human myotubes increases the angiogenic potential of SkM‐EVs. These angiogenic benefits coincided with increased anti‐oxidative capacity of recipient HUVECs. High PGC‐1α expression in skeletal muscle may prompt the release of SkM‐EVs that support vascular redox homeostasis and angiogenesis.

## Abstract (highlights)

- **What is the central question of this study?**
  Skeletal muscle extracellular vesicles likely act as pro‐angiogenic signalling factors: does overexpression of peroxisome proliferator‐activated receptor γ coactivator 1‐α (PGC‐1α) alter skeletal muscle myotube extracellular vesicle release, contents and angiogenic potential?
- **What is the main finding and its importance?**
  Overexpression of PGC‐1α results in secretion of extracellular vesicles that elevate measures of angiogenesis and protect against acute oxidative stress in vitro. Skeletal muscle with high levels of PGC‐1α expression, commonly associated with exercise induced angiogenesis and high basal capillarization, may secrete extracellular vesicles that support capillary growth and maintenance.

## 1 INTRODUCTION

Skeletal muscle fibres are surrounded by a microvascular network lined by endothelial cells (ECs) to ensure adequate exchange of gases and metabolites. Skeletal muscle regulates its microvascular network through the release of numerous pro‐ and anti‐angiogenic signalling factors (Gavin, 2009; Hellsten & Hoier, 2014; Olfert et al., 2016). Following acute exercise muscle fibres release robust levels of pro‐angiogenic factors that facilitate the formation of new blood vessels. Indeed, chronic exercise training is well‐understood to increase skeletal muscle capillary density in a variety of populations and training modalities (Gavin, 2009; Gavin et al., 2007; Verdijk et al., 2016).

Extracellular vesicles (EVs) are small membrane‐bound factors that facilitate autocrine, paracrine and endocrine signalling responses. EV cargo consists of varying RNAs, lipids and proteins. In skeletal muscle cells, extracellular vesicle (SkM‐EVs) cargo reflects the metabolic health of the cell of origin (Gao et al., 2021; Jalabert et al., 2021; Sullivan et al., 2022). For example, SkM‐EV contents differ between lean and obese individuals and are altered following 1 week of exercise training (Sullivan et al., 2022). Intramuscularly, SkM‐EVs may act as angiogenic signalling factors that maintain microvascular health and promote angiogenesis following exercise. We have found that SkM‐EVs increase aspects of angiogenesis – growth, migration and tube formation – in cultured ECs (Nie et al., 2019). Additionally, following a supraphysiological hypertrophy stimulus, SkM‐EVs are taken in by capillary ECs and are associated with increased gene expression of several angiogenesis‐regulating proteins (Murach et al., 2021). SkM‐EVs are not exclusively pro‐angiogenic, however, as stress‐induced cellular senescence causes muscle progenitor cells to secrete anti‐angiogenic and pro‐senescent EVs (Hettinger et al., 2021).

Skeletal muscle capillary density is tightly regulated to support the metabolic needs of muscle fibres. The primary determinants of muscle fibre capillary density are fibre size and oxidative capacity (mitochondrial volume) (Brodal et al., 1977; Gavin, 2009). Endurance exercise training stimulates muscle fibre mitochondrial biogenesis and increases capillarization to ensure oxygen delivery matches increased mitochondrial oxygen demand. The transcription factor peroxisome proliferator‐activated receptor γ coactivator 1‐α (PGC‐1α) is an important regulator of mitochondrial biogenesis and is activated following endurance exercise, leading to adaptive increases in skeletal muscle mitochondrial volume (Lin et al., 2002; Wu et al., 1999). PGC‐1α helps facilitate the link between mitochondrial biogenesis and angiogenesis following exercise in skeletal muscle. Overexpression of PGC‐1α increases skeletal muscle capillarization (Arany et al., 2008), while deletion prevents exercise‐induced angiogenesis (Chinsomboon et al., 2009). This is in part due to PGC‐1α‐regulated secretion of vascular endothelial growth factor (VEGF) and other pro‐angiogenic factors (Arany et al., 2008; Chinsomboon et al., 2009). Additionally, PGC‐1α has been shown to exert cytoprotective effects against increases in oxidative stress by upregulating anti‐oxidant enzymes and pathways (Aquilano et al., 2013).

It is well established that highly oxidative muscle fibres have more mitochondria and greater capillary density than highly glycolytic muscle fibres (Gavin, 2009). Highly oxidative muscle fibres secrete greater basal and exercise‐induced pro‐angiogenic factors to support microvascular homeostasis and growth (Annex et al., 1998; Cherwek et al., 2000; Gavin et al., 2007; Jia et al., 2020; Lloyd et al., 2003; Mounier et al., 2010). Consistent with this, we and others have recently found that primarily oxidative muscle tissue secretes more EVs than primarily glycolytic tissue in mice (Estrada et al., 2021; Nie et al., 2019). The intramuscular mechanisms regulating differences between highly oxidative and glycolytic muscle fibres are unknown. In the current investigation we hypothesized that overexpression of PGC‐1α in human myotubes would increase EV release and improve the pro‐angiogenic potential of SkM‐EVs in addition to being protective against an acute oxidative stress challenge in ECs.

## 2 METHODS

### 2.1 Human subject ethical approval

All protocols were approved by the Institutional Review Board at Purdue University (IRB no. 1406014975) in accordance with the *Declaration of Helsinki*, except for registration in a database. Written informed consent was obtained from all subjects prior to their participation in the study.

### 2.2 Isolation and culture of human muscle cells

Young (18–35 years), sedentary (<3 days/week regular exercise) and lean (body mass index <30 kg/m^2^) men and women were recruited to participate in this study (*n* = 8). Subjects were excluded if they had a history of cardiovascular or metabolic disease, regularly smoked or had a history of deep vein thrombosis. All subjects consented to participate in the study following written and verbal explanations of the intent and components of the study. Single muscle biopsies were taken from the vastus lateralis using the Bergstrom needle technique. Muscle samples were immersed in Dulbecco's modified Eagle's medium (DMEM) containing 10% fetal bovine serum (FBS), 1% streptomycin–penicillin (10,000 U/ml) and 1% sodium pyruvate (Sigma‐Aldrich, Oakville, ON, Canada). Samples were minced with scissors, then broken down enzymatically with a dispase/collagenase cocktail. Muscle mononuclear satellite cells (MuSC) were isolated using magnetically activated cell sorting with CD56^+^ microbeads (Miltenyl Biotec, Cambridge, MA, USA). MuSCs were grown in human skeletal muscle growth medium cat. no. 151‐500 (Cell Applications Inc., San Diego, CA, USA). Only early passaged cell (P2–P4) were used in this study. Upon reaching ∼90% confluence, MuSCs were differentiated into myotubes in differentiation medium, DMEM supplemented with 2% horse serum, 1% streptomycin–penicillin and 1% sodium pyruvate (ThermoFisher Scientific, Waltham, MA, USA), for control and viral overexpression experiments. Prior to EV collections, cells were incubated in DMEM supplemented with EV‐depleted 2% horse serum, 1% streptomycin–penicillin and 1% sodium pyruvate.

### 2.3 PGC‐1α adenoviral overexpression

On day 4 of differentiation, cells were treated with differentiation medium containing adenovirus for human PGC‐1α co‐expressing green fluorescent protein (GFP) (Ad‐PGC) or an adenovirus containing GFP (Ad‐GFP) for 48 h as previously described (Brault et al., 2010). Both viruses were at a final concentration of 7.5 × 10^6^ particle forming units (pfu)/ml. Following transfection, cells were washed with phosphate‐buffered saline (PBS) three times and cultured for an additional 48 h in EV‐depleted differentiation medium. Cells were collected and analysed for gene and protein expression of PGC‐1α, to confirm transfection, and members of EV‐release pathways. Conditioned medium was saved for EV isolation.

### 2.4 Mitochondrial content

Mitochondrial content was measured via MitoTracker Green FM (Thermo Fisher Scientific), to assess the impact of Ad‐PGC‐1α on mitochondrial biogenesis in skeletal muscle myotubes. Following the 48 h viral incubation, cells were incubated in the presence of MitoTracker Green fluorescent dye for 30 min at 37°C according to the manufacturer's instructions. After completion of the staining the cells were washed with PBS and fluorescence signal was measured using a fluorescence microplate reader at an excitation of 490 nm and emission of 516 nm.

### 2.5 RNA isolation, reverse transcription and real‐time qPCR

RNA was extracted from myotubes, myotube‐derived EVs, and human umbilical vein endothelial cells (HUVEC) with Trizol, following the manufacturer's instructions (Thermo Fisher Scientific). RNA concentration was measured via nanosight; samples were normalized and 1000 μg of first strand cDNA was generated by random hexamer primers with MMLV Reverse Transcriptase (Thermo Fisher Scientific). Real‐time PCR was performed using SYBR Green‐based chemistry on a CFS Connect (Bio‐Rad Laboratories, Hercules, CA, USA). Gene expression was determined using $2^{-\Delta \Delta C_{t}}$ relative quantification and normalized to 18S.

### 2.6 Protein extraction and immunoblotting

Myotube‐derived EVs were lysed and protein was collected in RIPA buffer, 50 mM NaF, 0.2 mM Na_3_VO_4_ and protease inhibitor cocktail (Thermo Fisher Scientific). Protein concentration was determined by BCA Assay (Thermo Fisher Scientific). Sample concentrations were normalized and 4× bromophenol blue loading buffer was added to each sample. Samples were incubated at 95°C for 5 min to linearize protein. Total protein (20 μg) was fractionated on SDS–polyacrylamide gels (10%), transferred to polyvinylidene difluoride membranes, blocked in 5% non‐fat milk, and incubated overnight at 4°C at a 1:1000 dilution in blocking buffer with primary anti‐ALIX (cat. no. 2171) and anti‐TSG101 (cat. no. 72312; Cell Signaling Technology, Danvers, MA, USA). Following incubation, membranes were washed and incubated in secondary anti‐rabbit or anti‐mouse IgG–horseradish peroxidase (Cell Signaling Technology) for 1 h at room temperature and protein detected using chemiluminescence (Bio‐Rad). Densitometric analysis was performed using Image Lab software (Bio‐Rad). Results were normalized to tubulin.

### 2.7 EV isolation and characterization

EVs were isolated from untreated and virally transfected myotubes at day 8 of differentiation. Cells were grown in 10 cm plates and a total of 20 ml of conditioned medium per group was collected for EV isolation. Cell conditioned medium was centrifuged at 2000 *g* for 10 min to remove dead cells and debris. EVs were isolated using a combined ultrafiltration‐size exclusion chromatography (UF‐SEC) method, recommended by the International Society of Extracellular Vesicles (Théry et al., 2018). Briefly, medium was concentrated from ∼15 ml to 500 μl using protein ultrafiltration tubes with 50 kDa filters (Thermo Fisher Scientific). Concentrated medium was passed through qEV SEC columns (IZON Science, Medford, MA, USA), designed to collect EVs in the 30–350 nm size range. The EV fraction was collected in sterile‐filtered PBS at a total volume of 1.5 ml. To determine EV concentration, a small portion (∼10%) of the EV supernatant was diluted in sterile‐filtered PBS to a final volume of 1000 μl and counted and characterized by size using a NanoSight LM10 Nanoparticle Tracking Analysis system (Malvern Panalytical, Westborough, MA, USA). The NanoSight is equipped with a 405 nm laser and a sCMOS camera. Following an auto‐setup, camera level and detection thresholds were set to 10, and all other settings were set to software auto settings. Particles were loaded into a syringe and pumped into the chamber, and five 30‐s videos were captured for analysis. Fresh sample was loaded into the chamber between captures. Particle concentration per ml and particle size distributions were analysed using NTA 2.3 software (Malvern Panalytical). EV concentration was further analysed by measuring total EV protein concentration using a BCA kit (Thermo Fisher Scientific). We have submitted all relevant data of our experiments to the EV‐TRACK knowledgebase (EV‐TRACK ID: EV220365).

### 2.8 Endothelial cell culture and EV treatments

HUVECs were obtained from a single donor (Cell Applications) and amplified in endothelial cell growth medium (EGM; Cell Applications). Cells were seeded into 96‐, 48, 24‐ or 6‐well plates depending on the assay. Untreated HUVECs were used as a control group and treatment groups consisted of EVs collected from untreated control myotubes (CON‐EVs), Ad‐GFP treated myotubes (GFP‐EVs) and Ad‐PGC treated myotubes (PGC‐EVs). Experimental assays were done in Human Endothelial Cell Basal media (Cell Applications; EBM) supplemented with 2% EV‐depleted FBS, unless otherwise noted. HUVECs were treated with EVs at a concentration of 10 μg/ml, as this has previously been an effective minimum EV dose for altering HUVEC function (Hettinger et al., 2021). EV treatment times depended upon the assay, ranging from 6 to 48 h.

Hydrogen peroxide treatments were performed to determine if PGC‐EV treatment improved recovery from oxidative stress. HUVECs were pretreated with 10 μg/ml GFP‐EVs or PGC‐EVs 3 h prior to being treated with 200 μM H_2_O_2_ for 1 h. Cells then recovered for 6–48 h depending on the assay being performed.

### 2.9 Cell proliferation and growth assays

5‐Ethynyl‐2′‐deoxyuridine (EdU) staining was performed to measure DNA replication, a proxy for cellular proliferation, following 48 h EV treatments in HUVECs. EdU (Carbosynth, San Diego, CA, USA) was added to culture medium and left for 15 h. Cells were then fixed in 4% paraformaldehyde (PFA; Sigma‐Aldrich) for 15 min. EdU staining was performed in 48‐well plates, with an initial cell density of 5000 cells/well. Incorporation of Edu into DNA was visualized using click‐it chemistry via the fluorescent dye TAMRA Azide 568 and total nuclei were visualized using 4′,6‐ diamidino‐2‐phenylindole (Thermo Fisher Scientific). Fluorescence cell images were taken with a CoolSnap HQ charge‐coupled camera (Photometrics, Tucson, AZ, USA) using a Leica DM6000 microscope (Wetzlar, Germany). Multiple images were taken at ×100 and ×200 magnification. Percentage proliferating cells was defined as EdU^+^ cells/total nuclei per field of view.

Cellular viability was assessed via a 3‐(4,5‐dimethylthiazol‐2‐yl)‐2,5‐diphenyltetrazolium bromide (MTT) assay. MTT assays were performed in 48‐well plates, with an initial cell density of 3,000 cells/well. Following a 48 h EV treatment, 25 μl of a 5 mg/ml MTT solution was added to each well for 2 h. Cells were then dissolved in 100 μl of DMSO and absorbance was measured at 570 nm.

### 2.10 Migration assay

HUVEC migration was analysed via scratch assay. Cells were grown to confluence on a 24‐well plate and gently scratched down the middle using a pipette tip (1000 μl) to detach cells. Wells were washed with PBS to remove detached cells and the medium was replaced with treatment EBM. Non‐control cells were treated with CON‐, GFP‐ or PGC‐EVs. Following a 12 h incubation, all cells were fixed in 4% PFA. Cells were imaged twice at ×25 magnification, immediately following the scratch, but prior to EV treatment, and after the 12 h incubation. Each post‐incubation image was compared to its pre‐treatment image for analysis. Cell migration was analysed by measuring the total area between the cells following the 12 h incubation. Total area between cells was measured using the wound healing size tool on ImageJ (National Institutes of Health, Bethesda, MD, USA), as previously described (Suarez‐Arnedo et al., 2020).

### 2.11 Tube formation assay

A tube formation assay was utilized to model the reorganization and tube forming processes of angiogenesis. The assay was performed in 96‐well plates precoated with Matrigel reduced growth factor basement membrane extract (Corning, Corning, NY, USA). Prior to the assay, 96‐well plates and pipette tips were chilled at −20°C and the Matrigel extract was thawed in ice at 4°C overnight. Each well was coated with 50 μl of Matrigel and incubated for 30 min at 37°C. During incubation, HUVECs were trypsinized and counted in serum‐free DMEM (Sigma‐Aldrich, St Louis, MO, USA). Cells were seeded onto the Matrigel‐coated wells at 12,000 cells per well and treated with EV in serum‐free DMEM. All treatments were done in duplicate. Cells were imaged after 6 h at ×25 magnification on a Leica DMi6000 microscope. Tube formation was measured using the angiogenesis analyser tool on ImageJ (NIH) as previously described (Carpentier et al., 2020). Total tubule length and number of tubes per field of view are reported.

### 2.12 Reactive oxygen species measurement

HUVEC intracellular reactive oxygen species (ROS) levels were measured using the cell‐permeant reagent 2′,7′‐dichlorofluorescin diacetate (DCFDA; Abcam, Waltham, MA, USA). DCFDA is a fluorescent dye that is oxidized to 2′,7′‐dichlorofluorescein (DCF) in the presence of hydroxyls, peroxyls and other ROS. HUVECs were seeded onto 24‐well plates at a density of 5,000 cells/well in 2% EBM. Cells were then treated with 10 μg/ml EV for 48 h. Immediately following the incubation, cells were washed once in PBS and incubated with DCFDA dissolved in phenol‐free DMEM for 30 min. Cells were then washed twice in PBS and fluorescence images were taken under low light conditions with a CoolSnap HQ charge‐coupled camera using a Leica DM6000 microscope. Three images were taken per well and exposure time was kept consistent for each image. Fluorescence intensity of each image was analysed using ImageJ.

### 2.13 Senescence‐associated β‐galactosidase staining

Senescence associated β‐galactosidase (SA‐β‐Gal) staining was performed using the Senescence‐Associated β‐Galactosidase Staining Kit (Cell Signaling Technology) at pH 6.0, as per the manufacturer's instructions. Following the 48‐h recovery from the H_2_O_2_ treatment, cells were fixed for 15 min and incubated overnight at 37°C, 5% CO_2_ with β‐Gal Staining Solution. Untreated HUVECs were used as controls for baseline senescence. Images were taken the following day in bright field and the percentage of senescent cells were those with β‐Gal positive staining relative to total cells in the field of view; three images were analysed per subject.

### 2.14 Statistical analysis

Analyses were conducted via either paired Student's *t*‐test or one‐way repeated measures ANOVA. Tukey's *post hoc* comparisons were run when appropriate. All analyses were done using GraphPad Prism (Version 9.2; GraphPad Software Inc., San Diego, CA, USA). *P* < 0.05 was considered statistically significant for all statistical sets. Data are presented as the mean ± SD.

## 3 RESULTS

### 3.1 PGC‐1α overexpression upregulates EV release pathways in myotubes, but does not impact EV release

**FIGURE 1:**
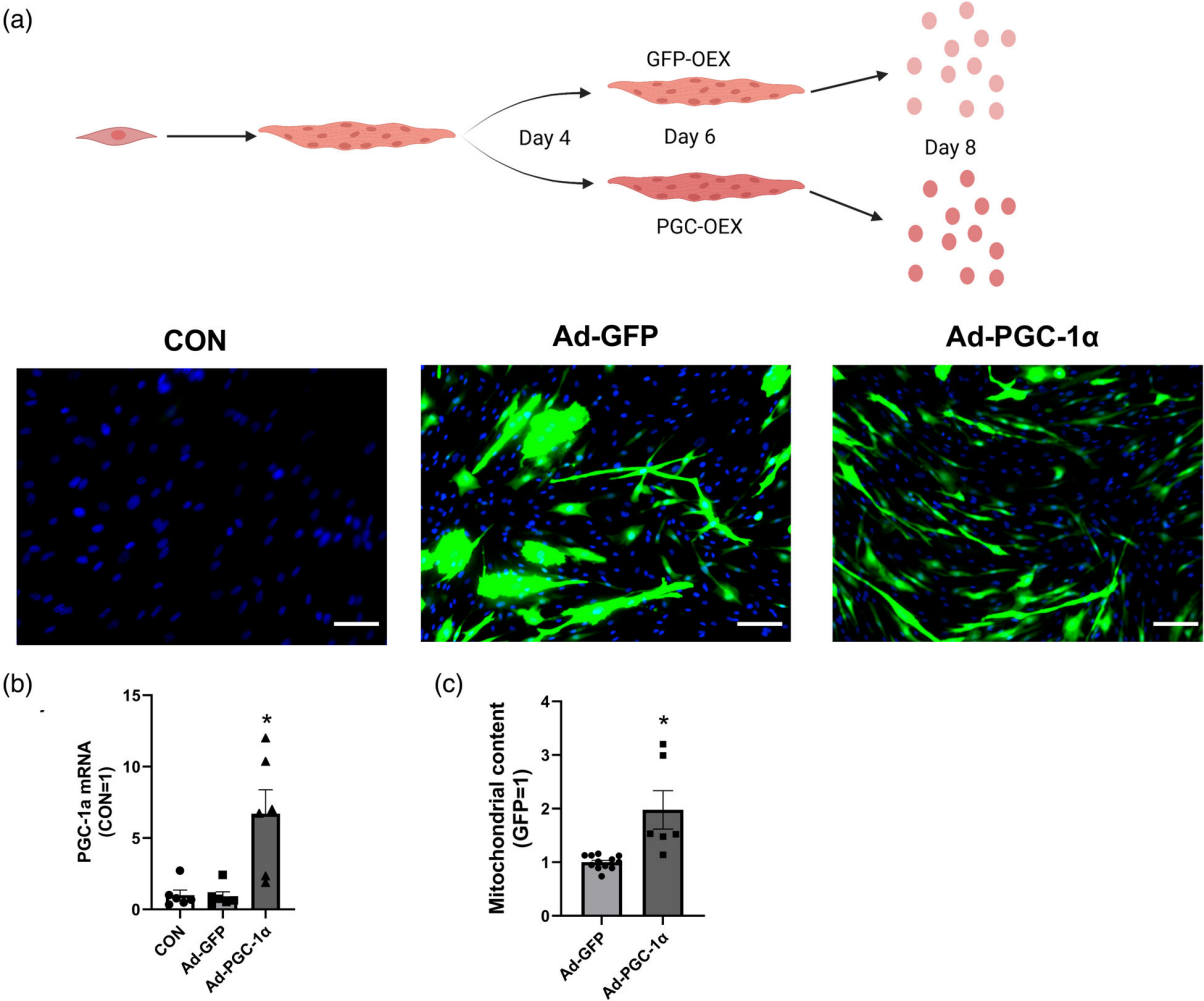
Adenoviral overexpression of PGC‐1α in human myotubes. (a) Schematic representation of Ad‐PGC and Ad‐GFP overexpression in differentiated human myotubes with representative immunofluorescence images demonstrating a lack of GFP in non‐viral control (CON) cell and GFP expression in Ad‐GFP and Ad‐PGC treated myotubes. (b) Gene expression of PGC‐1α. Gene expression is reported as fold change; CON = 1. (c) Mitochondrial content of adenovirus treated myotubes as measured by MitoTracker Green FM, reported as fold change; Ad‐GFP = 1. Mean + SD. *n* = 7–10/group. Scale bar: 200 μm. *P ≤ 0.05

Skeletal muscle myotubes were transfected with an Ad‐PGC‐1α that co‐expressed GFP or an Ad‐GFP viral control on day 4 of differentiation. Virus‐treated myotubes were compared to non‐viral control myotubes (CON). Both viral conditions induced strong GFP production in myotubes following transfection (Figure 1a). Ad‐PGC‐1α upregulated PGC‐1α gene expression more than seven‐fold compared to Ad‐GFP and CON (Figure 1b). There were no differences in PGC‐1α expression between Ad‐GFP and CON (Figure 1b). Ad‐PGC‐1α increased myotube mitochondrial content compared to the viral control (Figure 1c).

**FIGURE 2:**
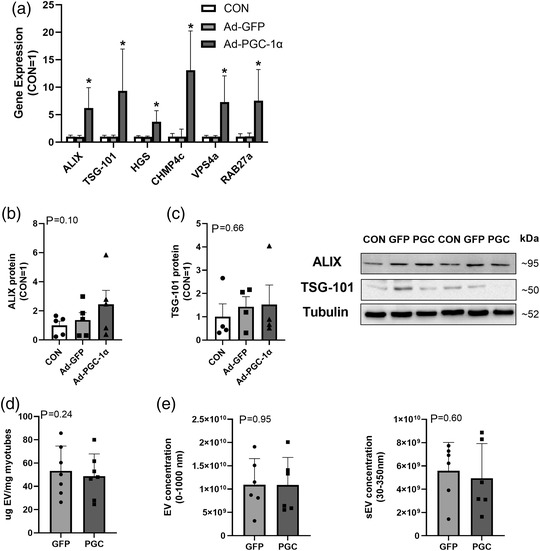
PGC‐1α overexpression upregulates gene expression of extracellular vesicle (EV) release pathway components but does not alter EV release. (a) Gene expression of EV release pathway components in myotubes. (b, c) Myotube protein expression of ALIX and TSG‐101 with representative blots. (d) EV release as measured by total protein concentration, normalized to original myotube total protein. (e) EV release as measured by nanosight analysis at 30–350 nm and 30–1000 nm size ranges, normalized to original myotube total protein. Gene and protein expression are reported as fold change; NVC = 1. Mean + SD. *n* = 6–8/group. *P ≤ 0.05

Multiple components of EV release pathways were measured, to determine if PGC‐1α overexpression altered EV release pathway gene expression. Compared to Ad‐GFP and CON, Ad‐PGC‐1α upregulated gene expression (Figure 2a) of hepatocyte growth factor‐regulated tyrosin kinase substrate (HGS) and tumour susceptibility gene‐101 (TSG‐101), and charged multivesicular body protein‐4 (CHMP4c), each a member of the endosomal sorting complex required for transport (ESCRT). Ad‐PGC‐1α also upregulated gene expression (Figure 2a) of apoptosis‐linked gene 2‐interacting protein X (ALIX), vacuolar protein sorting 4a (VPS4a) and RAB27a, which are all involved in facilitating EV release. There were no differences in expression of these genes between Ad‐GFP and CON (Figure 2a). Protein expression of ALIX and TSG‐101 did not different between Ad‐PGC‐1α, Ad‐GFP or CON myotubes (Figure 2b,c).

Following isolation via UF‐SEC, EV concentration was measured and normalized to total protein of the original myotubes. EV protein concentration did not differ between GFP‐EVs and PGC‐EVs (Figure 2d). There was no difference in total EV release or small EV (sEV; 30–350 nm) release as measured by nanosight analysis between GFP‐EVs and PGC1‐EVs (Figure 2e).

### 3.2 EVs from PGC‐1α‐overexpressing myotubes alter aspects of angiogenesis in ECs

**FIGURE 3:**
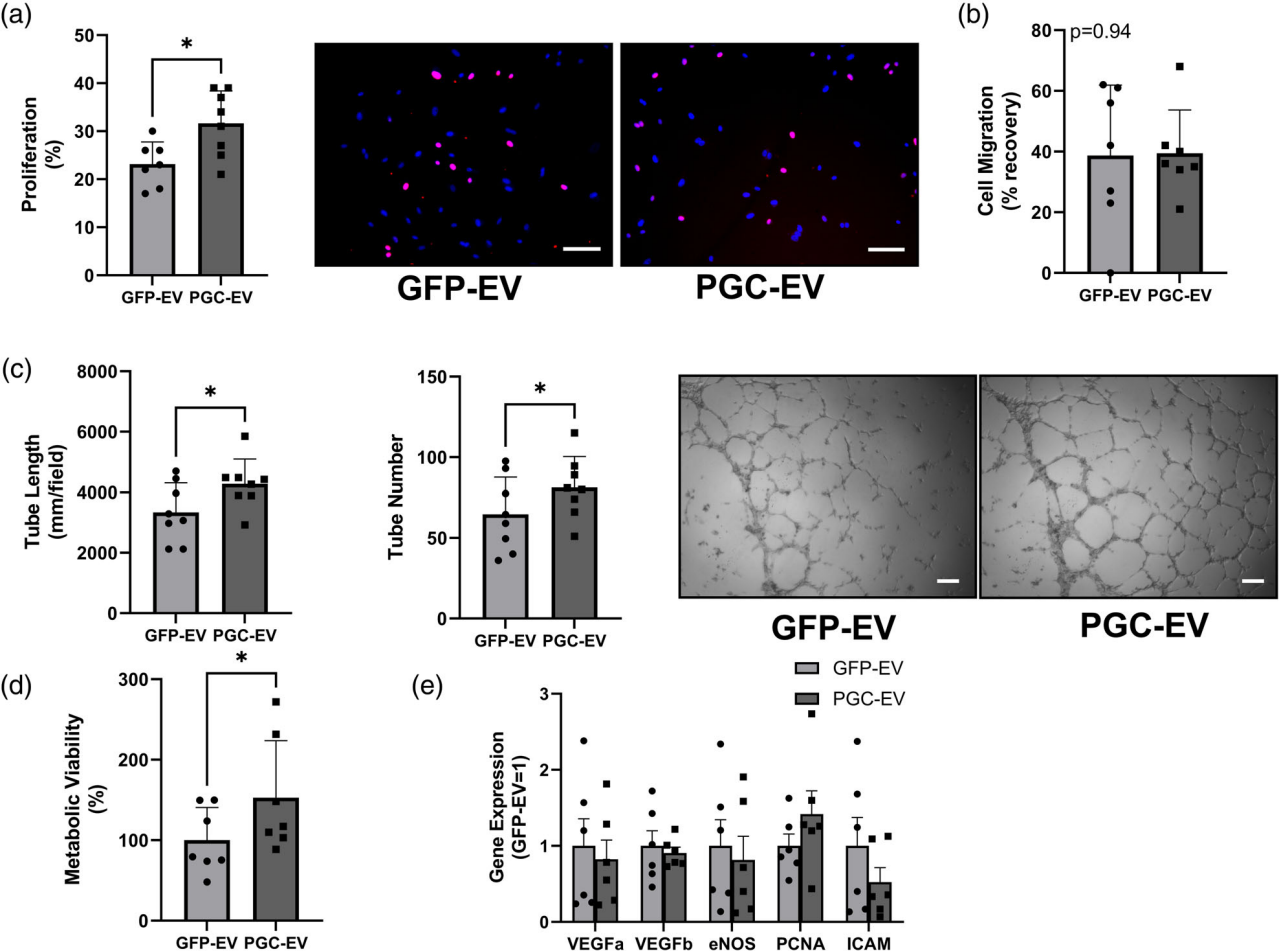
PGC‐EVs improve measures of angiogenesis in cultured endothelial cells. (a) Human umbilical vein endothelial cell (HUVEC) proliferation as measured by nuclear EdU incorporation, with representative images. (b, c) HUVEC migration (b) and tube formation (c) with representative images. (d) HUVEC metabolic viability as measured by MTT assay. (e) Gene expression of several angiogenesis regulating genes in EV‐treated HUVECs. Gene expression is reported as fold change; GFP‐EV = 1. Mean + SD. *n* = 7/group. Scale bar: 200 μm. *P ≤ 0.05

Human umbilical vein ECs were treated with GFP‐EVs and PGC‐EVs to test the hypothesis that PGC‐1α overexpressing myotubes secrete EVs with greater pro‐angiogenic potential than controls. PGC‐overexpressing EV treatment increased EC proliferation compared to GFP‐overexpressing EVs (Figure 3a). There was no difference in cell migration between the treatments (Figure 3b). ECs treated with PGC‐EVs formed more tubules and had greater overall tubule length than GFP‐EV‐treated cells (Figure 3c). PGC‐EV‐treated HUVECs had greater cellular viability as measured by an MTT assay, compared to GFP‐EV‐treated HUVECs (Figure 3d). Gene expression of several endothelial and angiogenesis regulating genes was measured following EV treatment. There was no difference between GFP‐EV‐ and PGC‐EV‐treated HUVECs in the expression of VEGFa, VEGFb, endothelial nitric oxide synthase (eNOS), proliferating cell nuclear antigen (PCNA) or intracellular adhesion molecule 1 (ICAM‐1) (Figure 3e).

### 3.3 EVs from PGC‐1α‐overexpressing myotubes alter redox homeostasis in ECs

**FIGURE 4:**
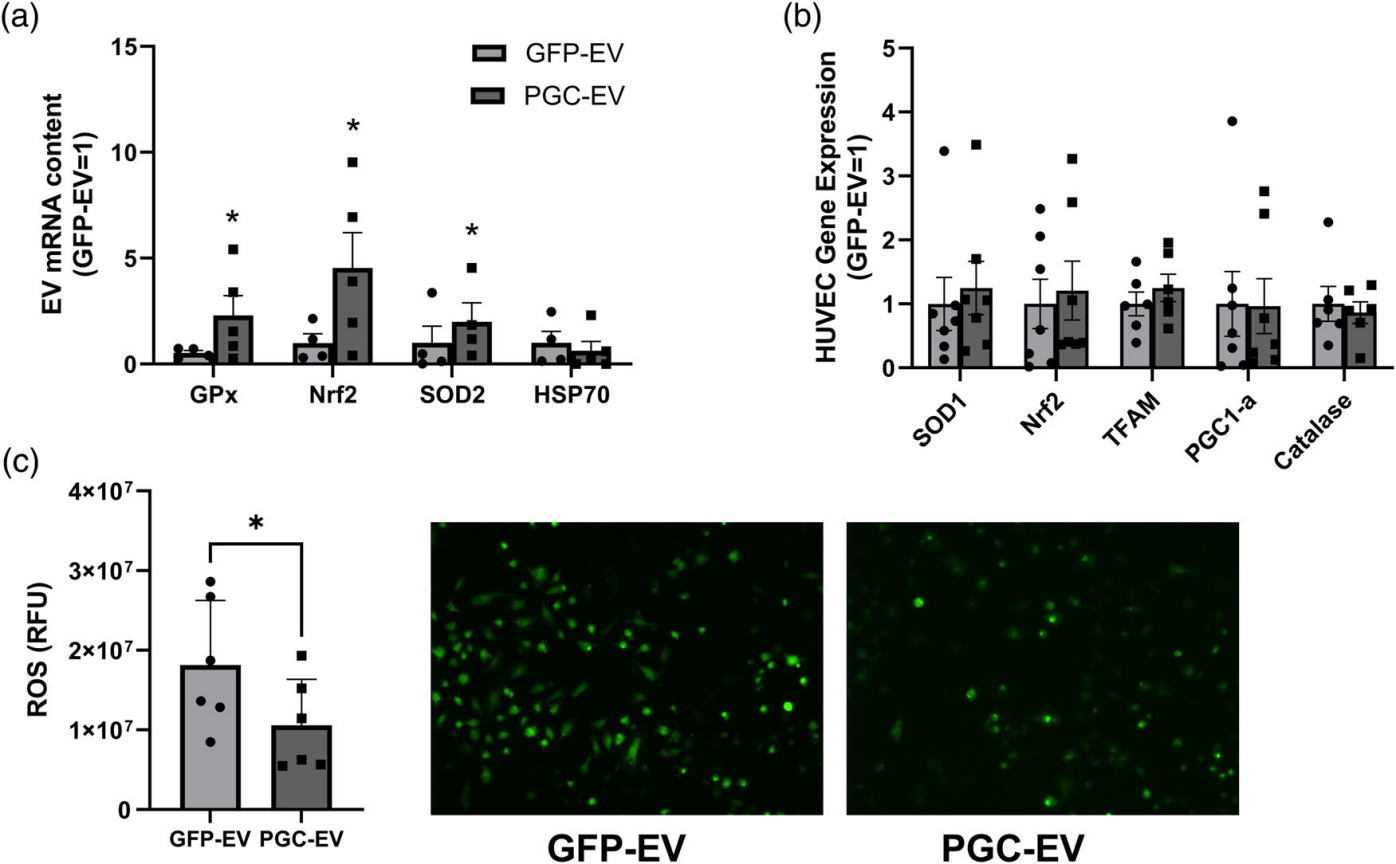
PGC‐EVs contain greater levels of antioxidative mRNA and decrease HUVEC reactive oxygen species (ROS) content. (a) mRNA contents of Gpx, Nrf2, HSP70 and SOD2 in GFP‐EVs and PGC‐EVs. (b) Gene expression of several anti‐oxidant and mitochondrial genes in EV‐treated HUVECs. (c) HUVEC ROS following 48 h EV treatments, with representative images. Gene expression is reported as fold change; GFP‐EV = 1. Mean + SD. *n* = 5–7/group. Scale bar: 200 μm. *P ≤ 0.05

RNA was isolated from EV fractions for analysis of select mRNA cargo. PGC‐EVs contained significantly greater amounts of superoxide dismutase 2 (SOD2), nuclear factor erythroid 2‐related factor 2 (Nrf2) and glutathione peroxidase (GPx) mRNA (Figure 4a). There was no difference in heat shock protein 70 (HSP70) transcripts between EV groups (Figure 4a). Expression of several mitochondrial and anti‐oxidative genes was measured following EV treatment. There was no difference between the treatments in the gene expression of superoxide dismutase 1 (SOD1), Nrf2, PGC‐1α, transcription factor A, mitochondrial (TFAM) and catalase (Figure 4b). HUVECs treated with PGC1‐EVs had lower levels of ROS (Figure 4c) compared to those that were GFP‐EV treated.

**FIGURE 5:**
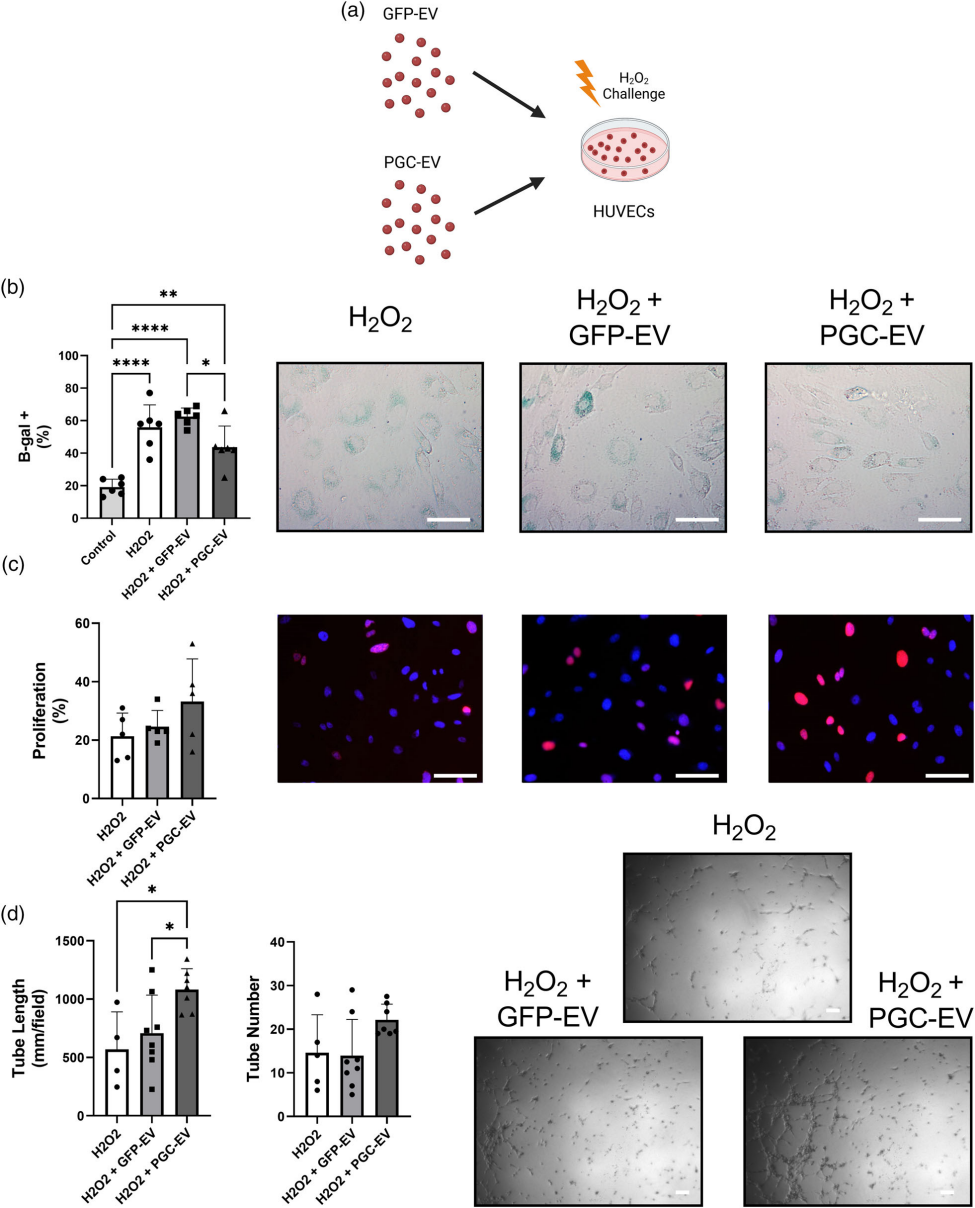
PGC‐EV treatment improves tube formation and reduces senescence following H_2_O_2_ treatment. (a) Schematic representation of EV‐H_2_O_2_ treatments.s (b) HUVEC senescence as measured by β‐galactosidase staining, with representative image. (c, d) HUVEC proliferation (c) and tube formation (d), with representative images. Mean + SD. *n* = 7–8/group. Scale bar: 50 μm. *P ≤ 0.05, **P ≤ 0.01, ***P ≤ 0.001, ****P ≤ 0.0001

HUVECs were pre‐treated with GFP‐EVs or PGC‐EVs before being incubated with 200 μM H_2_O_2_ to determine recovery following an oxidative stress challenge. PGC‐EV reduced cellular senescence 48 h following H_2_O_2_ treatment, compared to H_2_O_2_ control and GFP‐EV‐treated cells (Figure 5b). There was no difference in proliferation between the groups 48 h after H_2_O_2_ treatment (Figure 5c). Following the H_2_O_2_ treatment PGC‐EVs improved endothelial cell tube length compared to H_2_O_2_ control and GFP‐EVs, and there was a trend towards improved tube number in PGC‐EV‐ compared to GFP‐EV‐treated cells (*P* = 0.09) (Figure 5d).

## 4 DISCUSSION

The purpose of the current report was to determine whether PGC‐1α overexpression impacts SkM‐EV release and angiogenic potential. We present evidence that overexpression of PGC‐1α enhances the pro‐angiogenic potential of myotube‐derived EVs, but does not impact EV release. Additionally, we have identified that PGC‐EVs confer protective effects against acute oxidative stress that are not conferred by control GFP‐EVs. Together with our previous research (Nie et al., 2019), we have highlighted a novel pro‐angiogenic and cytoprotective role for muscle‐derived EVs that is strengthened in muscle presenting characteristics of an oxidative phenotype.

### 4.1 PGC‐1α overexpression in human myotubes alters EV contents but not release

Overexpression of PGC‐1α in myotubes upregulated PGC‐1α gene expression nearly eight‐fold, consistent with exercise‐induced increases (Pilegaard et al., 2003; Terada et al., 2002), and elevated myotube mitochondrial content. Acute exercise increases SkM‐EV release (Gao et al., 2021; Guescini et al., 2015) and it appears that EV release is regulated by intrinsic metabolic properties of muscle fibres and an oxidative phenotype (Estrada et al., 2021; Nie et al., 2019). Since PGC‐1α regulates the oxidative phenotype and is upregulated following exercise (Aquilano et al., 2013; Pilegaard et al., 2003; Russell et al., 2003; Terada et al., 2002; Zhang et al., 2017), we hypothesized that PGC‐1α overexpression would increase SkM‐EV release. In the current study, PGC‐1α overexpression did not alter SkM‐EV release, suggesting that PGC‐1α does not regulate the increase in SkM‐EV release seen in primarily oxidative, versus glycolytic, muscle tissue (Estrada et al., 2021; Nie et al., 2019). Interestingly, we observed robust increases in the expression of several genes involved in EV release pathways, but these transcriptional increases did not elevate EV release, nor were they correlated to myotube EV release (data not shown). Additionally, excess ALIX and TSG101 transcripts likely were not translated into protein as there were no differences in ALIX or TSG101 protein in Ad‐PGC‐1α‐treated cells. Transcript and protein levels are often discordant (Fukao, 2015; Gry et al., 2009), and frequently protein synthesis rate and post‐transcriptional regulation, rather than transcript number, is the main regulator of protein expression (Kristensen et al., 2013).

It remains to be determined precisely how EV release is regulated in skeletal muscle fibres. Skeletal muscle‐specific overexpression of Nrf2, a cytoprotective transcription factor regulated by PGC‐1α (Aquilano et al., 2013; Gureev et al., 2019), increases SkM‐EV release likely through increased muscle contractility (Gao et al., 2021). As there is considerable overlap in Nrf2 and PGC1‐α signalling and because PGC‐1α overexpression improves muscle contractile function (Gureev et al., 2019; Southern et al., 2019), it is possible that PGC‐1α indirectly regulates SkM‐EV release in vivo but not in non‐contractile myotubes. More work needs to be done to determine the intrinsic mechanisms that regulate SkM‐EV release.

PGC‐1α regulates pro‐angiogenic signalling factor transcription in muscle and is essential for exercise‐induced angiogenesis (Arany et al., 2008; Chinsomboon et al., 2009). Similarly, PGC‐1α regulates SkM‐EV contents, as PGC‐EVs contained greater levels of antioxidative and mitochondrial regulating transcripts (i.e., SOD2, Nrf2, Gpx). Notably, there was no increase in PGC‐1α mRNA in PGC‐EV‐treated HUVECs, indicating that the angiogenic benefits of PGC‐EVs were not due to cross transfer of Ad‐PGC via PGC‐EVs. While this is the first report to demonstrate that myotube PGC‐1α overexpression alters SkM‐EV contents, Gao and colleagues found that skeletal muscle‐specific overexpression of Nrf2 increases NAD(P)H: quinone oxidoreductase 1 (NQO1) and SOD2 protein in circulating EVs at rest and following exercise (Gao et al., 2021). PGC‐1α and Nrf2 have multiple overlapping mitochondrial and antioxidant regulating functions at rest and following exercise (Gureev et al., 2019). Taken together, these data suggest that cytoprotective transcription factors regulate SkM‐EV contents.

### 4.2 PGC‐1α overexpression enhances angiogenic potential of SkM‐EVs

We have previously shown C2C12‐EVs shuttle pro‐angiogenic miRNAs and increase angiogenesis in cultured ECs (Nie et al., 2019). Additionally, upregulation of pro‐angiogenic genes in muscle capillary cells is correlated to muscle‐derived EV uptake following a supra‐physiological exercise stimulus in rodents (Murach et al., 2021). The intrinsic metabolic properties of muscle cells appear to regulate the contents and signalling potential of SkM‐EVs (Estrada et al., 2021; Hettinger et al., 2021; Jalabert et al., 2021). Here we expand on that idea and provide evidence that PGC‐1α overexpression improves the pro‐angiogenic potential of SkM‐EVs. As hypothesized, PGC‐EVs elevated endothelial cell proliferation and tube formation compared to GFP‐EVs, but there was no impact on cell migration. Despite the alterations in proliferation and tube formation, GFP‐EV and PGC‐EV treatments did not alter EC gene expression of several pro‐angiogenic genes 48 h following EV treatment. The absence of improved migration following the scratch assay was unexpected, considering the tube formation assay contains a migratory aspect, with the cells migrating across the matrix before forming tubes. EC migration during angiogenesis is influenced by VEGF (Wang et al., 2011), so it is possible that the lack of VEGF upregulation in the current study is responsible for the lack of a difference in migration. In totality, we propose that skeletal muscle fibres with highly oxidative phenotypes release EVs that support a dense microvascular network.

### 4.3 PGC‐EVs impact metabolic and anti‐oxidant properties of ECs

Considering PGC‐1α overexpression increased SkM‐EV anti‐oxidative transcript contents, we wished to determine whether PGC‐EVs altered ROS in recipient ECs. PGC‐EV treatment reduced endothelial ROS 48 h after treatment. During exercise, muscle generates high levels of ROS that have several adaptive signalling functions including increasing PGC‐1α expression (Gureev et al., 2019; Powers et al., 2020; Scheele et al., 2009), leading to mitochondrial biogenesis, enhanced oxidative metabolic capacity and compensatory increases in anti‐oxidant enzymes (Schnyder & Handschin, 2015). Additionally, exercise‐induced ROS production and improved anti‐oxidant capacity in other tissues contribute to several exercise‐induced adaptations including angiogenesis (Dworakowski et al., 2008; Frey et al., 2009; Le Moal et al., 2017). EV‐mediated improvements in anti‐oxidant signalling have been observed in several tissues, including skeletal muscle (Gao et al., 2021). C2C12 EVs increase expression of SOD2 and elevate H_2_O_2_ levels in cultured ECs, indicating increased dismutation of superoxide to H_2_O_2_ (Nie et al., 2019). Acute exercise elevates circulating EV content of the extracellular superoxide dismutase 3 (SOD3) protein, restoring the angiogenic potential of circulating EVs in diabetic mice through improved anti‐oxidant capacity in recipient ECs (Abdelsaid et al., 2022). In the current report, PGC‐EVs appeared to increase endothelial cell anti‐oxidative capacity, as evidenced by lower ROS levels. Importantly, lower ROS did not blunt measures of angiogenesis. We postulate that an increase in PGC‐1α in skeletal muscle may result in production of EVs that can improve redox homeostasis and anti‐oxidative capacity of neighbouring tissues such as the microvasculature.

### 4.4 PGC‐EVs protect against ROS‐induced endothelial senescence and angiogenic dysfunction

To determine if EVs were protective against high levels of oxidative stress, cells were pretreated with GFP‐EVs and PGC‐EVs prior to an H_2_O_2_ challenge. Highly oxidative cellular environments can reduce the angiogenic capability of ECs and increase development of premature senescence (Cai & Harrison, 2000; Erusalimsky & Skene, 2009; Shosha et al., 2018). Cells pretreated with PGC‐EVs had greater tube formation and lower incidence of cellular senescence compared to GFP‐EVs. Importantly, the protective effects of EVs were dependent on PGC1‐α overexpression in muscle as GFP‐EVs did not alter the HUVEC response to oxidative stress compared to the H_2_O_2_ control. This suggests PGC‐EVs improve the capacity for ECs to prevent against acute oxidative stress and reduce development of a dysfunctional phenotype. Protective effects of SkM‐EVs against acute oxidative stress have been observed before, as muscle stem cell‐derived EVs reduce H_2_O_2_‐induced mitochondrial dysfunction in muscle myotubes (Shuler et al., 2020).

The precise mechanism through which PGC‐EVs protected ECs against acute oxidative stress are unclear. PGC‐EVs transported more antioxidative transcripts to ECs than GFP‐EVs, but this did not lead to differences in expression of these genes in recipient ECs. It is possible that non‐mRNA EV contents were responsible for the cytoprotective effects of PGC‐EVs. Skeletal muscle PGC‐1α is cytoprotective in numerous dysfunctional states (Chan et al., 2014; Kang & Li Ji, 2012; Selsby et al., 2012), and PGC‐1α overexpression protects against age‐associated capillary loss and preserves expression of angiogenic factors in old animals (Yang et al., 2020). Future studies should focus on determining whether EVs play a role in the beneficial effects of skeletal muscle PGC‐1α overexpression in skeletal muscle dysfunction in vivo.

### 4.5 Conclusion

Skeletal muscle fibres are well‐known regulators of the muscle microvasculature. The metabolic properties (i.e., primarily oxidative vs. glycolytic) and mitochondrial volume of skeletal muscle fibres correlate to basal capillary density and exercise‐induced angiogenesis in skeletal muscle. Mitochondria‐rich, highly oxidative skeletal muscle fibres release greater quantities of pro‐angiogenic factors, such as VEGF and EVs, than less capillarized glycolytic fibres (Estrada et al., 2021; Gavin, 2009; Nie et al., 2019). PGC‐1α expression is elevated following exercise and leads to transcriptional cascades that enhance mitochondrial biogenesis and support angiogenesis (Chinsomboon et al., 2009; Russell et al., 2003). The current results support the notion that SkM‐EVs can be pro‐angiogenic (Nie et al., 2019), with the delineation that PGC‐1α overexpression enhances the angiogenic properties and alters the contents of SkM‐EVs. Additionally, we demonstrate that PGC‐EV treatments can protect cultured ECs from acute oxidative stress. Future work should consider whether SkM‐EVs from PGC‐1α‐overexpressing muscle can act therapeutically to improve skeletal muscle microvascular dysfunction.

## AUTHOR CONTRIBUTIONS

C.K.K. designed experiments, collected and analysed data, and wrote the manuscript. TPG designed experiments and edited/contributed to the manuscript. D.M., A.Y., B.P.S. and L.C.B. contributed to data collection, experimental design, and manuscript review. J.J.B. and S.K. contributed to experimental design, data analysis, and manuscript review. All authors have read and approved the final version of this manuscript and agree to be accountable for all aspects of the work in ensuring that questions related to the accuracy or integrity of any part of the work are appropriately investigated and resolved. All persons designated as authors qualify for authorship, and all those who qualify for authorship are listed.

## CONFLICT OF INTEREST

None.

## Supporting information

Statistical Summary Document

## Data Availability

Raw data can be made available from the corresponding author upon reasonable request. All relevant data of our experiments has been submitted to the EV‐TRACK knowledgebase (EV‐TRACK ID: EV220365).
